# Report of the Prevalence of Diseases, &c., during the Month of December, 1865.—Broncho-Pneumonia Complicated with Diarrhœa.—Cerebro-Spinal Meningitis

**Published:** 1866-03

**Authors:** N. S. Davis


ARTICLE XII.

### REPORT ON THE PREVALENCE OF DISEASES, &c., DURING THE MONTH OF DECEMBER, 1865.—BRONCHO-PNEUMONIA COMPLICATED WITH DIARRHŒA.—CEREBRO-SPINAL MENINGITIS.

Read to the Chicago Medical Society by N. S. DAVIS, M.D., &c.

During the first twelve days of December, the weather was dry, mild, and pleasant, with winds chiefly from the north and west. Then came a severe snow-storm, immediately followed by seven or eight days of intense cold weather. About the 20th, the wind changed to the south, and it commenced thawing. It then continued warm and wet, the atmosphere being filled with mist and dampness, and the roads wet, until the evening of the 27th, when it again became freezing-cold, and remained moderately cold, clear, and dry until the end of the month.

Nothing unusual was noticed in regard to the prevalence of diseases during the first half of the month. Immediately following the extreme cold week that ended about the 20th of the month, catarrhal affections, consisting of slight inflammation of the mucous membrane of the nasal passages, fauces, and sometimes bronchial tubes, accompanied by rheumatic pains or general muscular soreness, became almost universally prevalent. Coincidently with these simple catarrhal affections, there occurred an unusual number of cases of pneumonia and broncho-pneumonia of a very persistent character, and, in a large proportion of the cases, coupled with typhoid looseness of the bowels. The cases of broncho-pneumonia were much more frequent among children than adults, especially children under five years of age. It also prevailed most in the same parts of the city that presented the greatest prevalence of diphtheria and typhoid fever during the two preceding months. Many of the cases commenced with the ordinary symptoms of simple bronchitis, namely, frequent and harsh cough; soreness in the chest; slight general febrile action, generally increased at night; from two to four intestinal evacuations daily, usually thin, green, and sometimes mucous; with coarse mucous rhonchus all over the chest. After five or six days, the respirations would become much more short and frequent; the tongue and mouth more dry; the skin more hot and dry; the pulse more sharp and frequent; the cough shorter and more distressing; the child often giving a moan or grunt with each expiration; the mind dull and sometimes drowsy; the bowels more loose; the urine often scanty and turbid; and the chest, both anteriorly and posteriorly, at the lower part giving a well-marked sub-crepitat râle, and some dulness on percussion. These symptoms generally persisted from one to two weeks, giving the patient, during the latter part of the time, a strongly-marked typhoid aspect, and then slowly declined, though many cases among the poorer classes of the people terminated fatally. In the cases of recovery, the convalescing stage was protracted and tedious.

During the same part of the month, there came under my observation four cases of cerebro-spinal meningitis, all in young persons; and an unusual number of cases of neuralgia, chiefly in the nerves of the face and neck.

During the last of the extreme cold days preceding the 20th of the month, I was consulted in regard to several cases of dizziness and a feeling of numbness in the head, in two instances amounting to temporary unconsciousness. All these cases were in persons who had been directly exposed to the cold, though, in some instances, very briefly. One, a female, past the middle period of life, had gone only one or two blocks, to a grocery store, and on her return fell unconscious in the street, from whence she was carried to her house, and in a short time recovered, though there remained a sense of confusion in the head when assuming an erect position for several days. No febrile reaction occurred in these cases. For several successive winters I have noticed similar disturbances in the functions of the brain in connection with extreme cold weather, especially in old people; and a simultaneous increase in the number of cases of convulsions and cerebral irritations in young children.

On the 27th of the month, the weather again became clear, dry, and moderately cold, and remained so until the end of the month, with a noticeable diminution of the catarrhal and pneumonic cases. The gross mortality for the month of December, as reported by the Health Officer, was 333; and for the whole year 1865, 3633; which, in a population of 180,000, would be equal to one death for every 49 inhabitants.

It should have been mentioned, that about the middle of the month of December, the water supplied for domestic use throughout the city, by the hydraulic works, became greatly impregnated with small fish, large numbers of which died in the water pipes, and, until past the middle of the succeeding month, kept the water so impregnated with the decaying fish as to render its taste and smell decidedly offensive. This was the only deviation from the usual local sanitary conditions of the city. Whether the use of such water had any influence in producing the persistent typhoid character presented by the principal diseases prevalent during that period, and in developing the few attacks of cerebro-spinal meningitis and erysipelas that occurred during the last part of the month, we leave each member of the Society to judge for himself.

All of which is respectfully submitted.

